# EIF4G1 Is a Potential Prognostic Biomarker of Breast Cancer

**DOI:** 10.3390/biom12121756

**Published:** 2022-11-26

**Authors:** Kun Li, Guangqing Tan, Xin Zhang, Weiyu Lu, Jingyi Ren, Yuewen Si, Enoch Appiah Adu-Gyamfi, Fangfang Li, Yingxiong Wang, Biao Xie, Meijiao Wang

**Affiliations:** 1 Department of Physiology, College of Basic Medicine, Chongqing Medical University, Chongqing 400016, China; 2 Department of Genetics, Development and Cell Biology, Iowa State University, Ames, IA 50011, USA; 3 Joint International Research Laboratory of Reproduction, Development of the Ministry of Education of China, School of Public Health and Management, Chongqing Medical University, Chongqing 400016, China; 4 Department of Biostatistics, School of Public Health and Management, Chongqing Medical University, Chongqing 400016, China

**Keywords:** breast cancer, prognosis, EIF4G1, immune infiltration, immunohistochemistry

## Abstract

Background: Breast cancer (BRCA) is one of the most common cancers in women worldwide and a leading cause of death from malignancy. This study was designed to identify a novel biomarker for prognosticating the survival of BRCA patients. Methods: The prognostic potential of eukaryotic translation initiation factor 4 gamma 1 (EIF4G1) was assessed using RNA sequencing (RNA-seq) data from The Cancer Genome Atlas (TCGA) and Gene Expression Omnibus (GEO) as training cohort and validation set, respectively. The functional enrichment analysis of differentially expressed genes (DEGs) was performed. The relationship between EIF4G1 and tumor microenvironment (TME) was analyzed. Immunotherapy responses were explored by the immunophenoscores (IPS) and tumor immune dysfunction and exclusion (TIDE) score. The Connectivity Map (CMap) was used to discover potentially effective therapeutic molecules against BRCA. Immunohistochemistry (IHC) was applied to compare the protein levels of EIF4G1 in normal and cancer tissues and to verify the prognostic value of EIF4G1. Results: BRCA patients with increased expression of EIF4G1 had a shorter overall survival (OS) in all cohorts and results from IHC. EIF4G1-related genes were mainly involved in DNA replication, BRCA metastasis, and the MAPK signaling pathway. Infiltration levels of CD4^+^-activated memory T cells, macrophages M0, macrophages M1, and neutrophils were higher in the EIF4G1 high-expression group than those in the EIF4G1 low-expression group. EIF4G1 was positively correlated with T cell exhaustion. Lower IPS was revealed in high EIF4G1 expression patients. Five potential groups of drugs against BRCA were identified. Conclusion: EIF4G1 might regulate the TME and affect BRCA metastasis, and it is a potential prognostic biomarker and therapeutic target for BRCA.

## 1. Introduction

Female breast cancer (BRCA) accounts for 24.5% of all new cancer cases and 15.5% of all cancer-associated death cases. It ranks as the one-sixth contributor and the fifth leading cause of cancer mortality worldwide [1]. Besides, it is one of the most common cancers among Chinese women [2]. In 2020, BRCA was diagnosed in approximately 2.3 million women, among whom 685,000 died [1,3]. Further, 18.4% of the BRCA cases were found in China, and this is the largest number of all global BRCA cases [3]. In the United States, BRCA is expected to account for approximately one third of all new cancer diagnoses in 2022 [4]. Histological stratification of BRCA is mainly based on the expression of progesterone receptor (PR), estrogen receptor (ER) and human epidermal growth factor receptor 2 (HER2). This is fundamental to BRCA classification [5,6]. There are Luminal A, Luminal B, HER2-enriched, basal-like, and normal-like subtypes of BRCA [7,8,9]. Currently, the primary treatment options for BRCA are radiation therapy, hormone therapy, chemotherapy, and surgery. Different drug combinations and targeted treatment have also proven to be good medical methods for BRCA [10]. Even though BRCA treatment has improved significantly, the associated endocrine resistance remains a huge challenge [11]. Due to this, patients with other metastatic diseases suffer poor prognosis [12]. Several immune-associated prognostic biomarkers of BRCA have been identified. However, many of them lack experimental verification while others were investigated with smaller sample sizes [13,14,15]. Hence, a novel and reliable immune-related biomarker identified via a rigorous experimental investigation of a large sample size is urgently needed.

Eukaryotic translation initiation factor 4 gamma 1 (EIF4G1) expression is increased in different types of cancers [16,17,18,19]. Upregulated EIF4G1 was found to be correlated with poor prognosis of nasopharyngeal carcinoma [16] and ovarian cancer [17]. Besides, increased EIF4G1 could promote the formation of tumor emboli by facilitating the translation of IRES-containing p120 mRNAs [18]. Cancer cells depend on cap-dependent translation to meet the demand for tremendous protein synthesis, which starts at 5’cap (m7GTP). Translation in eukaryotes is majorly regulated at the step of initiation on mRNA through the eukaryotic initiation factor 4F (EIF4F) components. EIF4F, mainly regulated by RAS/MAPK and PI3K/mTOR signaling pathways [20,21], is composed of EIF4A (an ATP-dependent RNA helicase), EIF4G (a scaffold protein), and EIF4E which binds m7 GTP cap [19,22]. The EIF4G family includes EIF4G1, EIF4G2, and EIF4G3. Among these isoforms, EIF4G1 is the most abundant (>85%) [23]. It has been reported that the elevated expression of EIF4G2 and m7G methylation-related genes, including EIF4G3, is closely associated with poor prognosis in hepatocellular carcinoma patients [24,25]. In previous studies, the expression of EIF4G1 was reported to be increased in BRCA. However, it has not been reported whether EIF4G1 is a prognostic biomarker for BRCA.

In this study, we systematically investigated the prognostic value of EIF4G1 in BRCA, determined the association of EIF4G1 with tumor-infiltrating immune cells (TIICs) and immunotherapy response, and screened for potential drugs against BRCA. External cohorts (including GSE88770 and GSE42568) and immunohistochemistry (IHC) were employed to validate our results. The correlation between tumor microenvironment (TME) and EIF4G1 was performed via ESTIMATE, CIBERSORT algorithms, and GEPIA.

## 2. Methods and Materials

### 2.1. Data Collection and Preprocessing

The mRNA expression profiles and corresponding clinical characteristics of BRCA patients were obtained from the UCSC website (https://xenaBRCAowser.net//, accessed on 11 March 2022). A total of 1083 female primary tumor patients were screened and used as the training set. Two gene expression arrays, GSE88770 (containing 117 tumor samples) and GSE42568 (containing 104 tumor samples and 17 normal samples), both based on the GPL570 platform, were downloaded from the Gene Expression Omnibus (GEO) (https://www.ncbi.nlm.nih.gov/geo/, accessed on 30 April 2022), and were selected as the external validation set. Patients without survival data were removed. After merging the tumor samples in the validation cohort and removing batch effects, we adjusted and normalized the mRNA expression data of the two microarray datasets.

### 2.2. Functions and Expression Analyses of EIF4G1 in Pan-Cancer

Cancer single-cell state atlas (CancerSEA) (http://biu.edu.cn/CancerSEA/, accessed on 31 May 2022) is the first database dedicated to decode 14 distinct functional states (including metastasis, stemness, invasion, proliferation, angiogenesis, apoptosis, cell cycle, hypoxia, differentiation, inflammation, quiescence, DNA damage, and DNA repair) of 25 cancer types at single-cell resolution [26,27]. Functions of EIF4G1 in different cancer types are found in this database. Tumor Immune Estimation Resource (TIMER) (https://cistrome.shinyapps.io/timer/, accessed on 8 May 2022), a tool to systematically analyze immune infiltration of 10,897 cancer samples from 32 types of cancer, was generated to investigate the difference of EIF4G1 expression between human cancer samples and paired normal tissues [28,29,30].

### 2.3. The Expression Level of EIF4G1 in Normal and Tumor Tissues

The Human Protein Atlas (HPA) (https://www.proteinatlas.org/, accessed on 8 May 2022) is a comprehensive website for researchers to study the protein localization and levels in common human organs, tissues, and cells [31,32,33]. On this basis, we observed the protein levels of EIF4G1 in normal and tumor tissues. IHC images of EIF4G1 protein level in BRCA tissues were also taken.

### 2.4. The Prognostic Value of EIF4G1 for BRCA

According to the optional cut-off value of EIF4G1, samples in The Cancer Genome Atlas (TCGA) were classified into high-expression and low-expression groups. Kaplan–Meier (KM) survival analysis of all patients and the three subtypes of BRCA was conducted to assess differences of overall survival (OS) between the two groups. Stratified groups of TCGA were utilized to estimate the predictive ability of this prognostic index for patients in different clinical subgroups. The time-dependent receiver operating characteristic (ROC) curve was plotted to predict OS of BRCA patients. Univariate and multivariate Cox regression analyses were performed depending on the clinicopathological factors (age and pathological stage) of TCGA to confirm whether EIF4G1 could predict the survival of BRCA patients.

Two microarray datasets (GSE88770 and GSE42568) containing the information of 221 BRCA patients were selected to validate our results. The correlation between OS and EIF4G1 expression in BRCA patients was determined using the KM survival curve. GSE88770 verified the relevance of EIF4G1 to OS in the three subtypes of BRCA.

### 2.5. Enrichment Analysis

Student’s *t*-test was performed to screen the EIF4G1-related differentially expressed genes (DEGs). Gene Ontology (GO) terms and Kyoto Encyclopedia of Genes and Genomes (KEGG) pathways were analyzed to discover the functional roles of DEGs. False discovery rate (FDR) < 0.05 was considered statistically significant.

### 2.6. Immune Cells Infiltration, Immune Checkpoints, and Immunotherapy Response Estimation

The ESTIMATE algorithm (https://bioinformatics.mdanderson.org/estimate/, accessed on 14 May 2022) was utilized to calculate the stromal scores (SSs) and immune scores (ISs) of each tumor tissue in TCGA. Then, the correlations of EIF4G1with SSs and ISs were analyzed [34].

The CIBERSORT approach was applied to analyze the proportions of 22 TIICs via a normalized gene expression matrix (http://cibersort.stanford.edu, accessed on 10 August 2022) [35,36]. Meanwhile, the Wilcox test was applied to compare the immune infiltration differences between the EIF4G1 high- and low-expression groups. 

GEPIA (http://gepia.cancer-pku.cn/, accessed on 8 May 2022), an online database, provides researchers with customizable functionalities based on TCGA and GTEx data. The correlations between EIF4G1 and T cell exhaustion markers were visualized through a scatter plot [37].

The Cancer Immunome Atlas (TCIA) (https://tcia.at/, accessed on 16 November 2022) database was used to download the immunophenoscores (IPS) of BRCA [38]. The relationship between EIF4G1 and IPS was analyzed to predict immunotherapy sensitivity. Tumor immune dysfunction and exclusion (TIDE) score was calculated via TIDE algorithm (http://tide.dfci.harvard.edu/, accessed on 16 November 2022) to infer patients’ response to immune checkpoint blockade (ICB) treatment, whose main targets are PD-L1, PD-1, and CTLA4 [39].

### 2.7. Identification of Potential Therapeutic Compounds

The Connectivity Map (CMap) (https://clue.io/, accessed on 10 August 2022), a novel database used to study gene interactions and drugs, can be applied for discovering potentially effective molecules against certain diseases [40]. Through this database, we found five potential therapeutic compounds against BRCA using the CMap tool in the “query” module via the L1000 platform after recognition of 50 up- and 40 down-regulated genes as valid genes, which are EIF4G1-related. Five compounds with an enrichment score of ≤ 0 with the lowest scores were screened as candidate inhibitors.

### 2.8. IHC Staining Evaluation 

Formalin-fixed human BRCA tissue specimens of 4 μm thickness were purchased from Shanghai Superbiotek Pharmaceutical Technology Co., Ltd (Shanghai, China), including 80 pairs of tumor tissues and 80 non-cancerous breast tissues. Tissue sections were incubated at 60 °C for 12 h, then dewaxed in xylene and hydrated in gradient alcohol. Slides were placed in Tris-EDTA buffer (pH = 9.0) for antigen repair in a microwave. Endogenous peroxidase blocker was used to eliminate endogenous interference, and normal goat serum was used to block nonspecific antigens at room temperature. EIF4G1 polyclonal antibody (1:150, Proteintech, Wuhan, China) was used to probe the slices at 4 ℃ overnight. The sections were incubated with a secondary antibody (Zsbio, Beijing, China) under room temperature for 30 min. Then, the samples were stained with diaminobenzidine, counterstained with hematoxylin, and dehydrated with gradient alcohol. The IHC staining results were independently assessed by two observers who were unaware of the patients’ clinical information. Staining intensity was defined as 0 = negative staining, 1 = weak staining, 2 = moderate staining, and 3 = strong staining. The percentage of positive cells was defined as 0 = 0–5%, 1 = 6%–25%, 2 = 26%–50%, 3 = 51%–75%, and 4 = 75–100%. The final result was obtained by multiplying the staining intensity and the staining percentage. According to the optimal cut-off value of 3, staining score > 3 represented high expression of EIF4G1 and staining score ≤ 3 represented low EIF4G1 expression.

KM analysis was used to evaluate the prognostic value of EIF4G1 in BRCA. Paired differential analysis was performed to compare the expression of EIF4G1 in tumor tissues and non-cancerous breast tissues.

### 2.9. Statistical Analysis

The R software (version 4.0.4, https://www.r-project.org/, accessed on 6 March 2022) was used for the statistical analyses. The validation cohort was adjusted with the “limma” (version 3.46.0) and “sva” (version 3.38.0) packages. Differences in protein levels between different groups in the TCGA and the GSE42568 cohorts were determined with the Wilcoxon test and the student’s *t* test, respectively. KM survival curves, univariate and multivariate Cox regression analyses were performed using the “survival” (version 3.3.1) and the “survminer” (version 0.4.9) packages. The time-dependent ROC curve was plotted with the “timeROC” package (version 0.4). To identify the DEGs, we conducted Student’s *t*-test and FDR. SSs and ISs were conducted with the “estimate” packages (version 1.0.13). The “ggplot2” package (version 3.3.6) was employed to visualize the result of functional enrichment analysis. The CIBERSORT was implemented using “e1071” package (version 1.7.9). *P*-value < 0.05 indicated significant differences between groups. Patients who had no OS information were excluded from both data sets.

## 3. Results

### 3.1. Research Process

**Figure 1:**
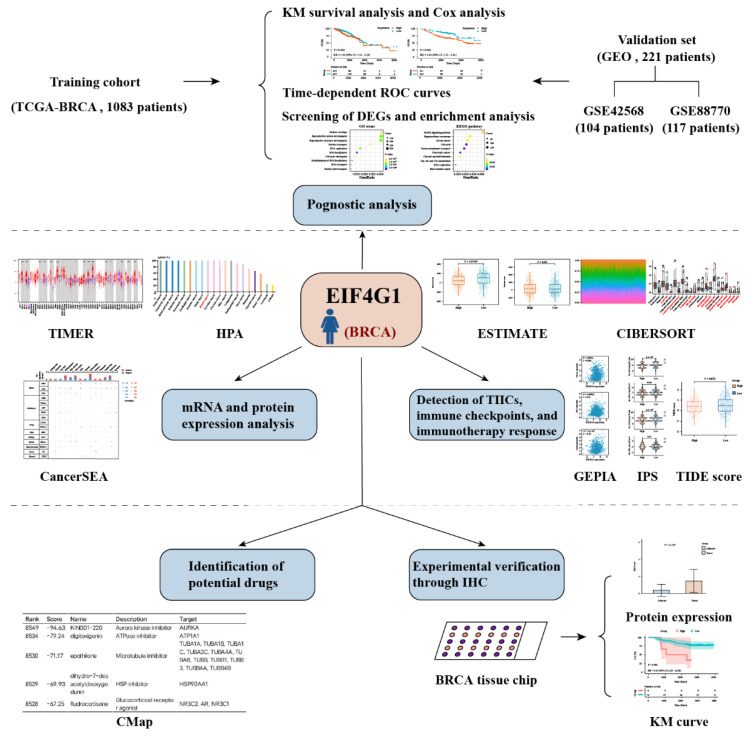
The workflow chart of the study.

The overall workflow diagram of this study is summarized in Figure 1. The training cohort comprised 1083 samples of TCGA while the meta-validation dataset was from 221 patients. The demographics and clinicopathological characteristics of these patients are displayed in Table 1.

### 3.2. Association of EIF4G1 with Pan-Cancer

**Figure 2:**
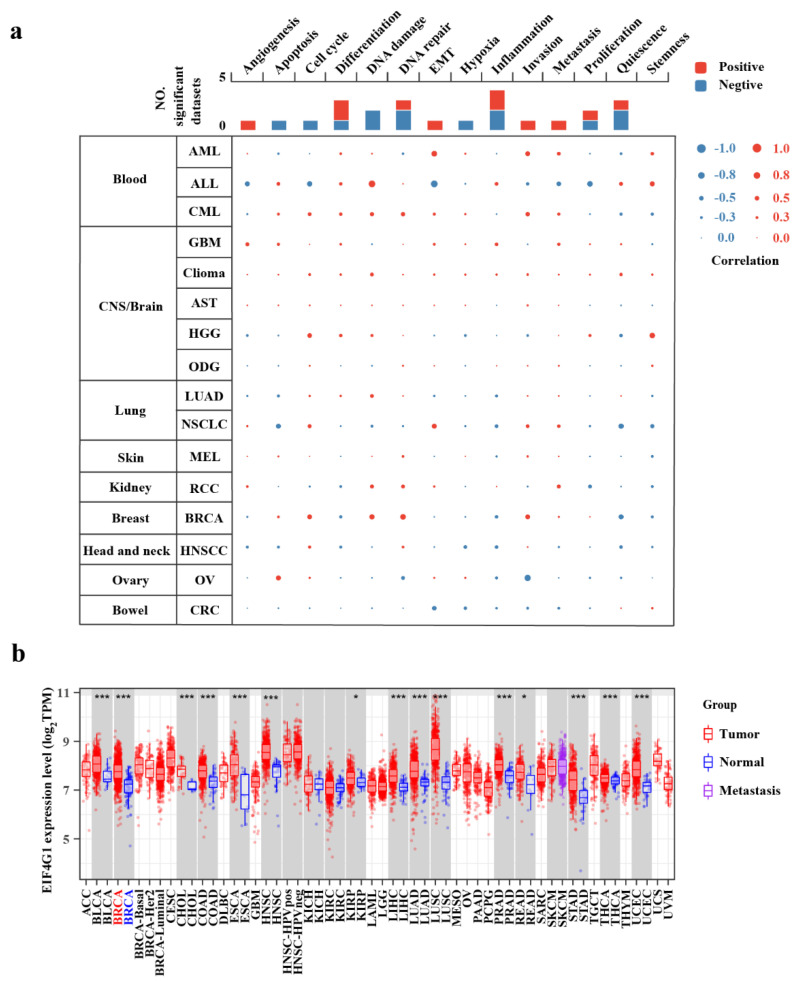
EIF4G1 is upregulated in pan-cancer and is involved in numerous processes. (**a**) Functional states of EIF4G1 and its association with 14 different types of cancers in the CancerSEA database; (**b**) The expression levels of EIF4G1 in different cancer types from the TCGA database, as analyzed with TIMER (* *p* < 0.05, *** *p* < 0.001).

The results with *p*-values and correlation were obtained from the CancerSEA single cell database. As the interactive bubble Chart shows, EIF4G1 was positively associated with DNA repair (*r* = 0.21, *p* = 0) and DNA damage (*r* = 0.20, *p* = 0) in BRCA (Figure 2a).

### 3.3. Upregulation of EIF4G1 in BRCA

The analyzed result of the TIMER database indicated that EIF4G1 mRNA level was significantly elevated in BRCA tissues compared to that of normal breast samples. Additionally, compared with adjacent normal tissues, EIF4G1 mRNA expression was observed to be increased in other 14 types of cancers, including bladder urothelial carcinoma, cholangiocarcinoma, colon adenocarcinoma, esophageal carcinoma, head and neck cancer, renal papillary cell carcinoma, lung adenocarcinoma, lung squamous cell carcinoma, prostate adenocarcinoma, rectum adenocarcinoma, stomach adenocarcinoma, thyroid carcinoma, uterine corpus endometrial carcinoma, and hepatocellular carcinoma (Figure 2b). 

**Figure 3:**
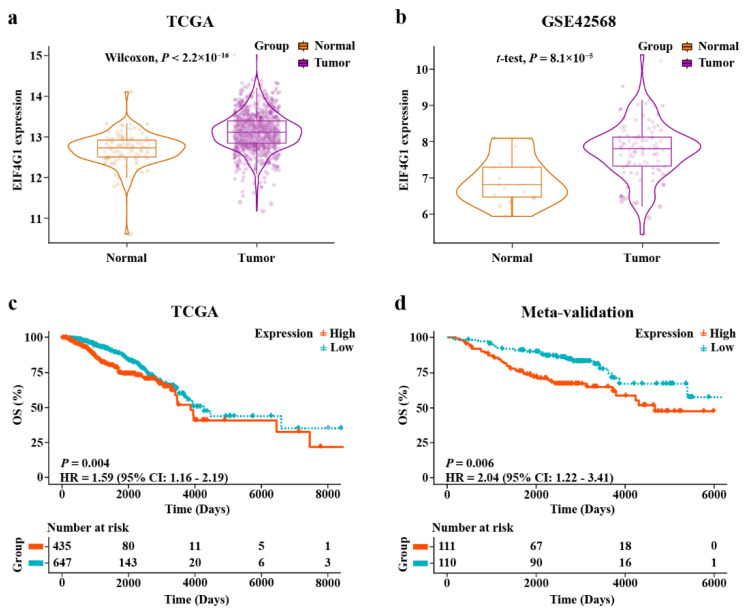
Upregulated EIF4G1 in two cohorts, and the results from KM survival analyses. (**a**,**b**) Different expression of EIF4G1 between breast cancer tissues and adjacent normal tissues in TCGA cohort and GSE42568; (**c**) KM survival analysis of high- and low-expression groups in TCGA-training cohort; (**d**) KM survival curve of the high- and low-expression groups in the meta-validation dataset.

EIF4G1 was found to be more significantly upregulated in the tumor tissues than in the paired adjacent normal tissues, in both the TCGA cohort (*p* < 2.2 × 10^–16^) and the GSE42568 cohort (*p* = 8.1 × 10^–5^; Figure 3a,b).

### 3.4. The Expression Level of EIF4Gl in HPA

HPA indicated that the protein level of EIF4G1 was higher in normal breast tissues and BRCA samples than in normal organs and tumor tissues (Figure S1a,b). IHC staining of BRCA tissues showed that EIF4G1 protein was mainly distributed in the cytoplasm. The staining was “moderately to strongly” positive (Figure S1c).

### 3.5. Functional Enrichment Analysis of EIF4G1

**Figure 4:**
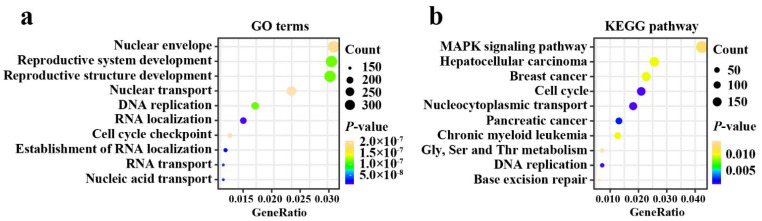
Functional annotations. (**a**) Top ten GO terms enrichment analysis of DEGs; (**b**) Top ten KEGG pathway enrichment analysis of DEGs. Gly, Glycine; Ser, serine; Thr, threonine.

The result of functional enrichment analysis showed that the number of enriched terms totaled 1237 (Table S1). The top 20 most significant GO functions and KEGG pathway analyses were sorted in ascending order of FDR. GO terms enrichment analysis revealed that the DEGs were primarily associated with DNA localization, DNA replication, and RNA transport (Figure 4a). The result of KEGG pathway analysis demonstrated that DEGs were mostly involved in MAPK signaling pathway, breast cancer, cell cycle, and DNA replication (Figure 4b).

### 3.6. Prognostic Performance of EIF4G1 in BRCA

The KM analysis of TCGA showed that BRCA patients with higher expression level of EIF4G1 had a short OS (HR = 1.59, *p* = 0.004; Figure 3c). In the three subtypes of BRCA, patients of HER2 positive BRCA had a poor OS when the expression of EIF4G1 was elevated (HR = 2.81, *p* = 0.02; Figure S2a–c). In addition, stratification analyses of the clinical features, including stage (Figure S3a,b) and age (Figure S3c,d), showed that patients in EIF4G1 high-expression group had short outcomes. The area under the curve (AUC) predictive values for three-, five-, and seven-year survival rates were 0.612, 0.615, and 0.546, respectively. This suggested that EIF4G1 had a certain capability to predict the prognosis of BRCA patients (Figure S4a). The results showed that EIF4G1 could predict the prognosis in patients at age >58 (three-year AUC = 0.640, five-year AUC = 0.611, seven-year AUC = 0.587). It could also predict their prognosis in the early stage (three-year AUC = 0.635, five-year AUC = 0.659, seven-year AUC = 0.590) and advanced stage (three-year AUC = 0.614, five-year AUC = 0.600, seven-year AUC = 0.472) of BRCA (Figure S4b–d).

Furthermore, univariate and multivariate Cox regression analyses were performed to confirm whether EIF4G1 could serve as an independent predictor of the survivability of BRCA patients. The univariate Cox regression analysis showed that age (*p* = 0.000285), stage (*p* = 1.21 × 10^–8^), and EIF4G1 (*p* = 0.00444) were prognostic factors for BRCA in the training cohort. The multivariate Cox regression analysis indicated that EIF4G1 is an independent prognostic indicator of BRCA (*p* = 7.86 × 10^−5^; Table S2). 

Our results were verified in the meta-validation dataset. As shown in d, upregulated EIF4G1 was correlated with poor prognosis (HR = 2.04, *p* = 0.006). The outcome for patients with increased EIF4G1 was unfavorable in the advanced grade subgroup (Figure S3e,f). The time-dependent ROC testified the predictive efficiency of EIF4G1 for BRCA (three-year AUC = 0.590, five-year AUC = 0.641, and seven-year AUC = 0.670) and for patients in advanced grade (three-year AUC = 0.567, five-year AUC = 0.621, and seven-year AUC = 0.649; Figure S4e,f). The multivariate analysis proved that EIF4G1 could serve as an independent predictor of unfavorable outcomes for BRCA cases (*p* = 0.0182; Table S2). In the GSE88770 cohort, patients of HER2 positive BRCA showed the same result as TCGA (*p* = 0.03; Figure S2d–f).

### 3.7. Analyses of Immune Infiltration, Immune Checkpoints, and Immunotherapy Response

**Figure 5:**
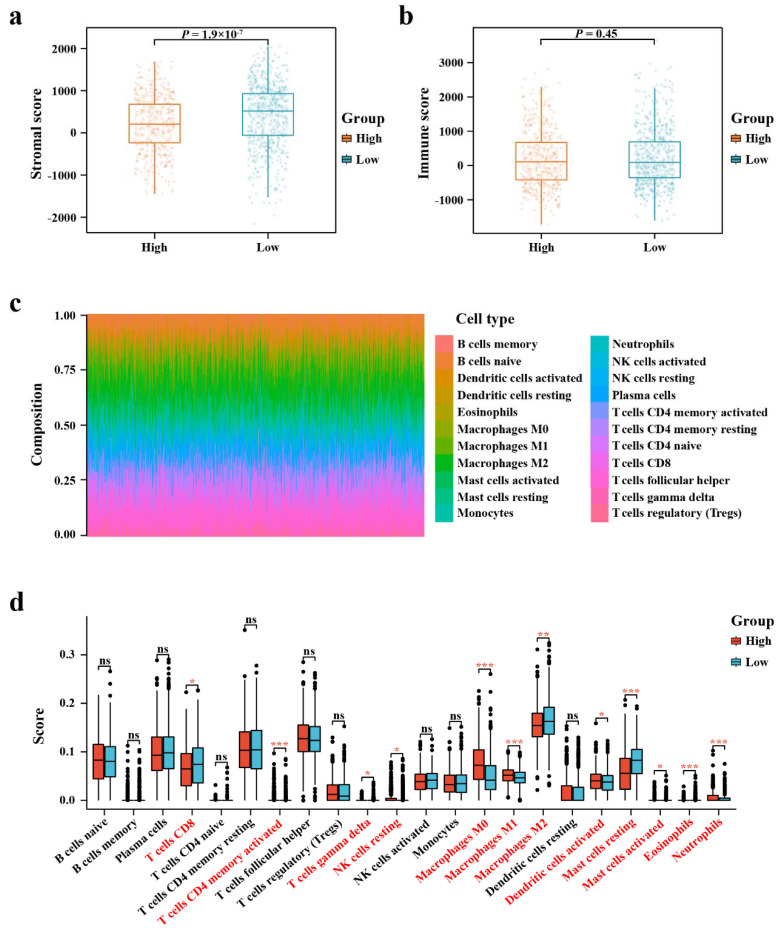
ESTIMATE scores distribution and TIICs analysis by CIBERSORT. (**a**, **b**) Distribution of ISs and SSs between EIF4G1 high- and low-expression groups; (**c**) A bar chart showing the difference in the proportion of 22 TIICs in the TME of BRCA; (**d**) A boxplot comparing the proportion of the 22 TIICs in the TME of BRCA between the high- and low-expression groups (* *p* < 0.05, ** *p* < 0.01, *** *p* < 0.001; ns, not significant).

Graphs from the ESTIMATE algorithm revealed the relevance between EIF4G1 and immune infiltration score. As shown in Figure 5a, the SSs in the high-expression group was significantly lower compared with that in the low-expression group. However, there was no difference in ISs between the high- and low-expression groups (Figure 5b).

The fractions of 22 kinds of immune lymphocytes in the TME of BRCA were performed by using the CIBERSORT algorithm (Figure 5c). Results of immune landscape conducted between the high- and low-expression groups indicated that proportions of CD4^+^ -activated memory T cells, macrophages M0, macrophages M1, and neutrophils were relatively higher in the EIF4G1 high-expression group than those in the EIF4G1 low-expression group, while infiltration levels of resting mast cells and eosinophils were lower in the EIF4G1 high-expression group (Figure 5d)**.**

**Figure 6:**
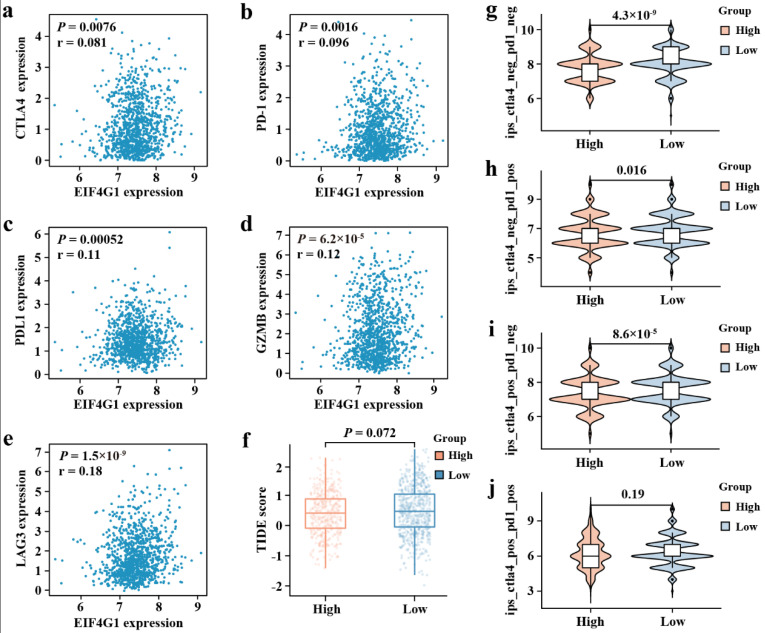
Correlation analysis of EIF4G1 to the immune checkpoint, TIDE score, and IPS. (**a**) CTLA4, (**b**) PD-1, (**c**) PDL1, (**d**) GZMB, and (**e**) LAG3 from GEPIA. (**f**) TIDE score between EIF4G1 high- and low-expression expression groups. (**g**) The IPS, (**h**) IPS-PD-1/PD-L1/PD-L2, (**i**) IPS-CTLA4, and (**j**) IPS-PD-1/PD-L1/PD-L2 + CTLA4.

Using the GEPIA database, we explored the association between immune checkpoints and EIF4G1. The results indicate that EIF4G1 is positively associated with cytotoxic T-lymphocyte-associated protein 4 (CTLA4), programmed cell death 1 (PD-1), programmed cell death 1 ligand 1 (PDL1), granzyme B (GZMB), and lymphocyte activating 3 (LAG3) (*p* < 0.001; Figure 6a–e).

Taking into account the positive correlation of EIF4G1 to the immune checkpoints, we then investigated the association between immune checkpoint inhibitors (ICIs) and EIF4G1. IPS between EIF4G1 high- and low-expression groups showed that patients of low EIF4G1 expression had a higher IPS of anti-PD-1 and anti- CTLA4 immunotherapy, which suggested a better immunotherapy response (Figure 6g–j). There was no significant correlation between TIDE score and EIF4G1 level (Figure 6f).

### 3.8. Screening for Potential Small Molecules Drugs

We found that five groups of drugs with highly negative enrichment scores might be beneficial in treating BRCA (Table 2). They were aurora kinase inhibitors, ATPase inhibitors, microtubule inhibitors, heat-shock protein (HSP) inhibitors, and glucocorticoid receptor agonists.

### 3.9. IHC Experimental Verification

**Figure 7:**
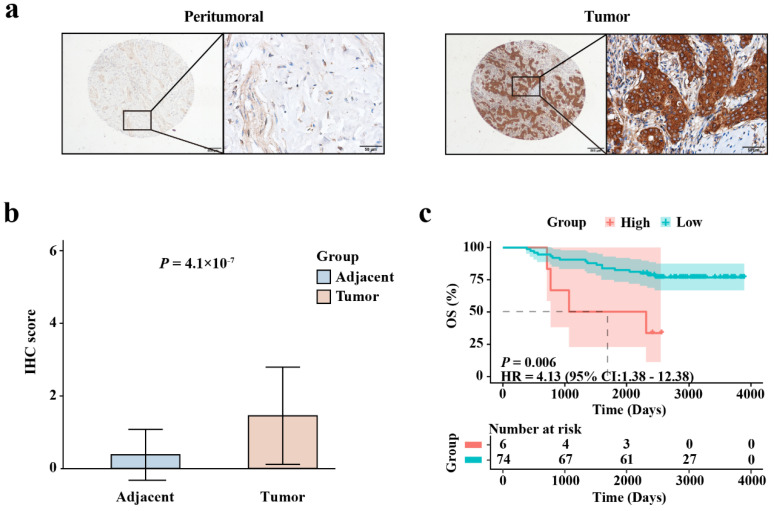
IHC staining validating the expression level and prognostic value of EIF4G1 in BRCA. (**a**) Representative IHC images of EIF4G1 in tumors and non-cancerous breast tissues. Scale bars were 500 μm and 50 μm; (**b**) Expression level of EIF4G1 in BRCA tissues and non-cancerous breast tissues; (**c**) Differences in OS between EIF4G1 high- and low-expression groups, as determined with the KM curve.

Representative IHC images of EIF4G1 in BRCA and non-cancerous breast tissues are shown in Figure 7a. EIF4G1 was mainly localized in the cytoplasm of the specimens, and the staining was predominant in the tumor tissues. This is consistent with the results in the HPA database. Figure 7b confirmed that the protein level of EIF4G1 was higher in the cancer specimens than in the non-cancerous breast tissues (*p* = 4.1 × 10^−7^). KM survival analysis (Figure 7c) suggested that patients with high expression of EIF4G1 had a short OS (HR = 4.13, *p* = 0.006). These results are consistent with the analysis of the training cohort and meta-validation set. There was no significant difference in the expression level of EIF4G1 among different clinical characteristics (Table S3).

## 4. Discussion

In this study, we found that EIF4G1 was upregulated in various solid tumors. Wu et al. [19] also found that EIF4G1 expression was commonly increased in tumors. The protein level of EIF4G1 in BRCA tissues was higher than that in the adjacent normal breast samples [18], which was consistent with our results from IHC, TCGA, and GSE42568 cohorts. Additionally, increased expression of EIF4G1 was associated with a short OS of BRCA patients. Subgroups analyses revealed that EIF4G1 was related to patients’ poor outcomes at age > 58 and stage in TCGA and advanced grade in the meta-validation set. EIF4G1 could still effectively predict the OS of BRCA patients with diverse clinical features, which was consistent with other reports [41,42]. Results from multivariate Cox regression analysis showed that EIF4G1 was an independent prognostic marker of BRCA. External validation cohort and IHC experiment verified the reliability and stability of this prognostic marker. Taken together, EIF4G1 may be an effective prognostic biomarker of BRCA.

EIF4G1 was mainly localized in the cytoplasm of the specimens, and the staining was predominant in the tumor tissues. This indicated that the protein level of EIF4G1 was higher in cancerous breast tissues than in non-cancerous breast tissues. This was consistent with the information in the HPA database. KM survival analysis suggested that patients with high expression of EIF4G1 had a short OS. The above results were consistent with the analysis of the training cohort and the meta-validation set.

Our GO and KEGG enrichment analyses showed that EIF4G1-related genes were primarily involved in cell cycle, DNA localization, DNA replication, RNA transport, nucleocytoplasmic transport, and the MAPK signaling pathway. Michelle Badura et al. [22] observed that increased expression of EIF4G1 could promote cell survival, DNA repair, and DNA damage response. This corroborates the results obtained from CancerSEA. EIF4G1 was found to promote cell growth, proliferation, and differentiation. It was also found to prevent autophagy and apoptosis [22,43]. The MAPK pathway may partly increase EIF4E phosphorylation to drive progression and metastasis through several mechanisms [20]. Recently, phosphorylated EIF4E was found to promote BRCA cell invasion through regulating the expression of IL-33 in fibroblasts [44]. As described above, EIF4G1 may play an important role in the tumorigenesis of BRCA. Therefore, our results suggested that EIF4G1 might affect the proliferation and metastasis of BRCA cells through regulating the MAPK signaling pathway.

We conducted immune microenvironment analysis and found that EIF4G1 expression level was associated with TIICs and immune checkpoints. TIICs was shown to have prognostic roles in various cancers, including BRCA [45,46,47]. Higher ICB treatment response rates were observed in IPS and TIDE score, which demonstrated that patients with higher expression of EIF4G1 could have a better response to immunotherapy. Therefore, EIF4G1 might be an effective factor for foretelling the effect of immunotherapy in BRCA patients. However, the exact mechanisms of association between EIF4G1 and TIICs in the TME and immune checkpoints need to be elucidated in well-designed studies. There was a significant difference in the proportion of SSs between high- and low-expression groups. Many cancer researchers have reported that SSs play important roles in the progression, metastasis, and therapy resistance of tumors [48,49]. There was no difference in ISs between the high- and low-expression groups, and this may be due to the different proportions of the various immune cells between the two groups. The estimation method indicated that the expression of EIF4G1 may be related to the progression and metastasis of BRCA.

Interestingly, two drugs, epothilone and digitoxigenin, have already been observed to inhibit BRCA metastasis. Epothilone was found to promote cancer cell death in the treatment of human cancers [50,51]. Digitoxigenin is a digitalis aglycone [52]. An early report found that digitalis could be an inhibitor for BRCA [53].

There are limitations in this work. Firstly, we could only minimize batch effects during cohort validation, rather than completely removing them. Moreover, this cohort study is retrospective. Hence, prospective studies are needed to verify our findings.

## 5. Conclusions

EIF4G1 was found to be more expressed in tumor tissues than para-cancerous breast tissues. Poor prognosis was significantly correlated with the high expression of EIF4G1 in breast cancer. Furthermore, EIF4G1 might regulate the proliferation and metastasis of BRCA cells. EIF4G1 showed significant association with TIICs, immune checkpoints, and IPS. Taken together, EIF4G1 has the potential to be an independent prognostic biomarker of short OS of BRCA patients and therapeutic target for treating BRCA.

## Figures and Tables

**Table 1:** Clinical features of BRCA patients in the TCGA and meta-validation dataset.

Clinical Features	TCGA (*n* = 1083)	GSE42568 (*n* = 104)	GSE88770 (*n* = 117)
**OS** Alive Dead	933 (86.15%) 150 (13.85%)	69 (66.35%) 35 (33.65%)	89 (76.07%) 28 (23.93%)
**Age** ≤58 >58	545 (50.32%) 538 (49.68%)	56 (53.85%) 48 (46.15%)	- -
**Grade** G1 G2 G3	- - -	11 (10.58%) 40 (38.46%) 53 (50.96%)	13 (11.11%) 96 (82.05%) 7 (5.98%)
**PR status** Positive Negative Indeterminate	688 (63.53%) 342 (31.58%) 4 (0.37%)	- - -	79 (67.52%) 37 (31.62%)
**ER status** Positive Negative Indeterminate	795 (73.41%) 238 (21.98%) 2 (0.18%)	- - -	106 (90.60%) 11 (9.40%) -
**HER2 status** Positive Negative Indeterminate	161 (14.87%) 557 (51.43%) 12 (1.11%)	- - -	7 (5.98%) 108 (92.31%) -
**Stage** Stage I Stage II Stage III Stage IV Stage X	182 (16.81%) 613 (56.60%) 247 (22.81%) 19 (1.75%) 14 (1.29%)	- - - - -	- - - - -
**T stage** T1 T2 T3 T4	279 (25.76%) 624 (57.62%) 138 (12.74%) 39 (3.60%)	- - - -	- - - -
**N stage** N0 N1 N2 N3	512 (47.28%) 356 (32.87%) 119 (10.99%) 76 (7.02%)	- - - -	- - - -
**M stage** M0 M1	901 (83.19%) 21 (1.94%)	- -	- -

OS, overall survival; ER, estrogen receptor; PR, progesterone receptor; HER2, human epidermal growth factor receptor 2.

**Table 2:** A list of screened compounds with highly negative enrichment scores.

Rank	Score	Name	Description	Target
8549	−94.63	KIN001-220	Aurora kinase inhibitor	AURKA
8534	−79.24	Digitoxigenin	ATPase inhibitor	ATP1A1
8530	−71.17	Epothilone	Microtubule inhibitor	TUBA1A, TUBA1B, TUBA1C, TUBA3C, TUBA4A, TUBA8, TUBB, TUBB1, TUBB3, TUBB4A, TUBB4B
8529	−69.93	Dihydro-7-desacetyldeoxygedunin	HSP inhibitor	HSP90AA1
8528	−67.25	Fludrocortisone	Glucocorticoid receptor agonist	NR3C2, AR, NR3C1

## Data Availability

The datasets presented in this study can be found in online repositories. The names of the repository/repositories and accession number (s) can be found in the article/Supplementary Material.

## Supplementary Materials

The following supporting information can be downloaded at: https://www.mdpi.com/article/10.3390/biom12121756/s1; Figure S1. EIF4G1 protein level in human cancers and normal tissues. (a) Protein levels of EIF4G1 in normal organs; (b) Protein levels of EIF4G1 in tumor tissues; (c) Representative IHC images of EIF4G1 expression level in BRCA tissues and normal samples; Figure S2. The KM survival curves of the three subtypes of BRCA. (a) Hormone receptor (HR) positive/HER2 negative, (b) HER2 positive, (c) triple-negative in the TCGA cohort. (d) HR positive/HER2 negative, (e) HER2 positive, (f) triple-negative in the GSE88770 cohort; Figure S3. KM survival stratification analyses of clinical characteristics pertaining to (a) early stage, (b) advanced stage, (c) age ≤58, (d) age >58, (e) early grade, and (f) advanced grade; Figure S4. The time-dependent ROC curve for 3-, 5-, and 7-year survival rates. (a) AUC predictive value of EIF4G1 in TCGA; (b) AUC predictive value of EIF4G1 for age >58, (c) early stage, (d) and advanced stage in TCGA; (e) AUC predictive value of EIF4G1 in meta-validation; (f) AUC predictive value of EIF4G1 for advanced grade in GEO; Table S1. GO and KEGG enrichment analyses results in TCGA; Table S2. Univariate and multivariate Cox analyses of prognostic factors in TCGA-training and meta-validation cohorts; Table S3. Clinical data of BRCA specimens in tissue microarray.
