# Supplementary material for: EIF4G1 Is a Potential Prognostic Biomarker of Breast Cancer
Source: Biomolecules. 2022 Nov 26;12(12):1756. doi: 10.3390/biom12121756 (PMC9776011; doi:10.3390/biom12121756)
Supplement: Supplementary file 1 [file biomolecules-12-01756-s001.zip › Figure S1-S4.pdf]

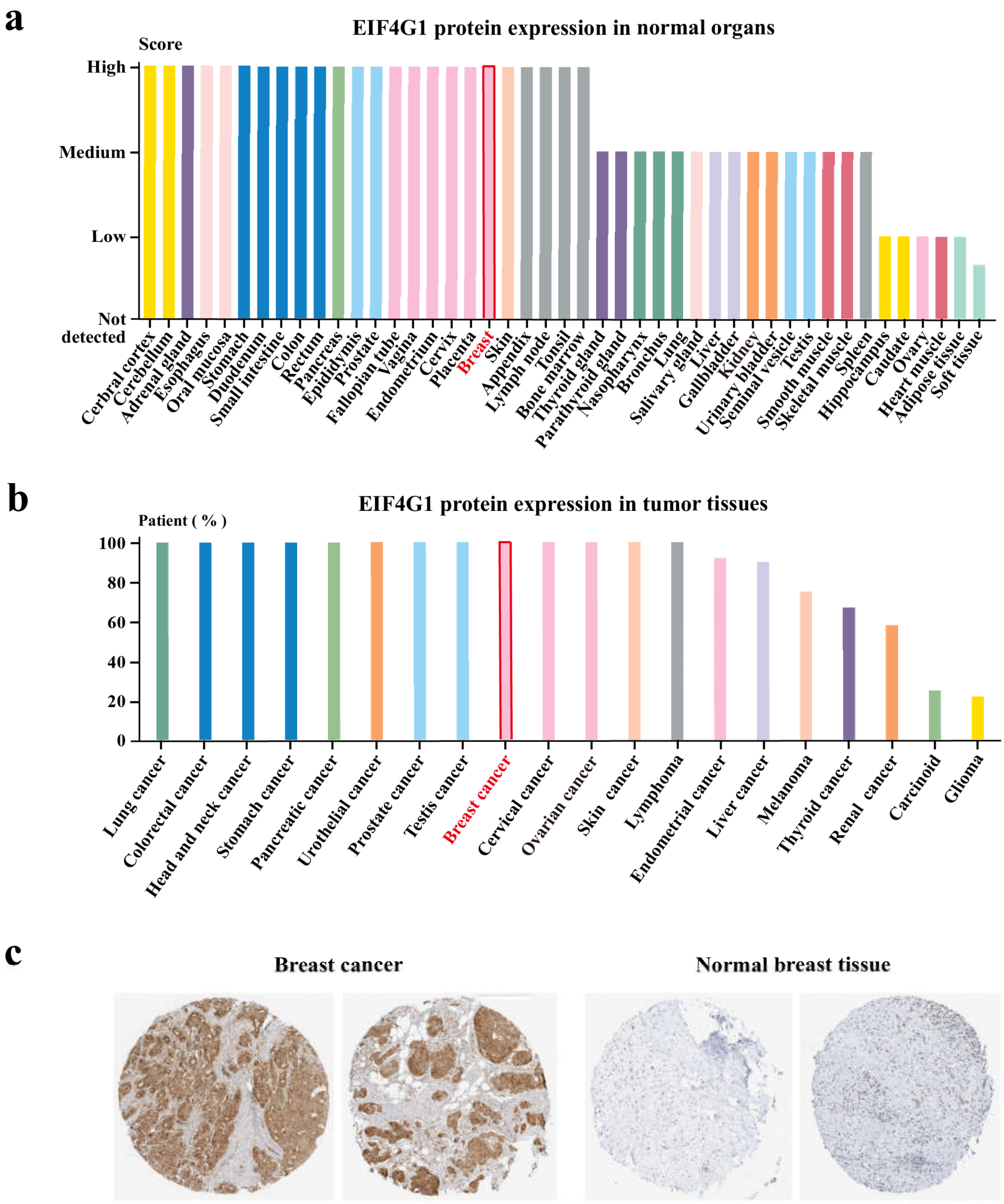

Figure S1. EIF4G1 protein level in human cancers and normal tissues. (**a**) Protein levels of EIF4G1 in normal organs; (**b**) Protein levels of EIF4G1 in tumor tissues; (**c**) Representative IHC images of EIF4G1 expression level in BRCA tissues and normal samples.

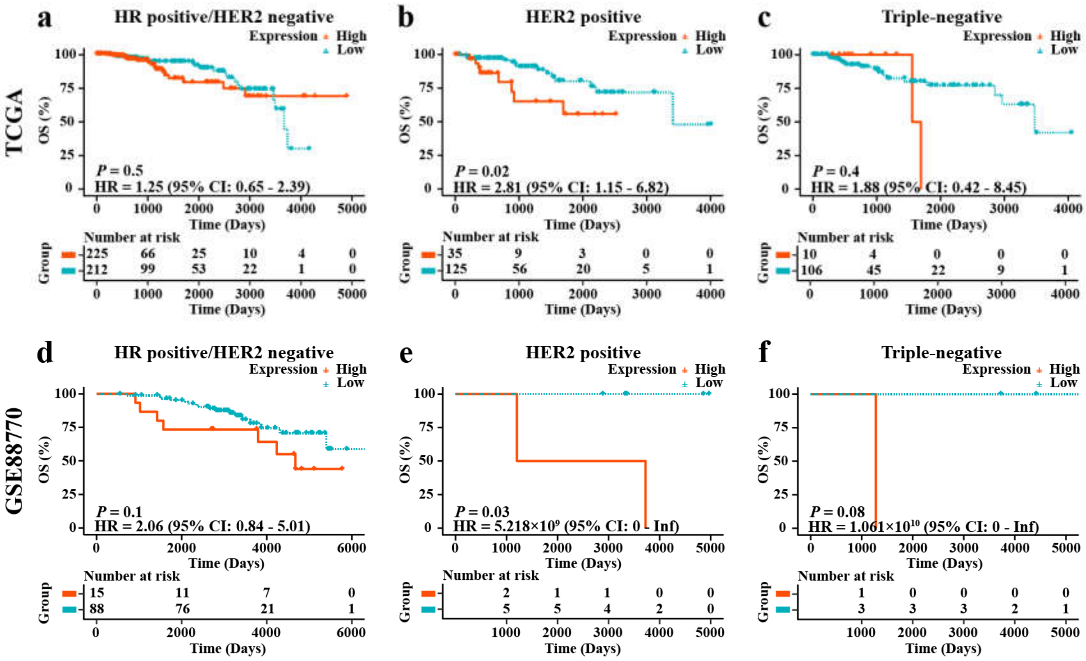

Figure S2. The KM survival curves of the three subtypes of BRCA. (**a**) Hormone receptor (HR) positive/HER2 negative, (**b**) HER2 positive, (**c**) triple-negative in the TCGA cohort. (**d**) HR positive/HER2 negative, (**e**) HER2 positive, (**f**) triple-negative in the GSE88770 cohort.

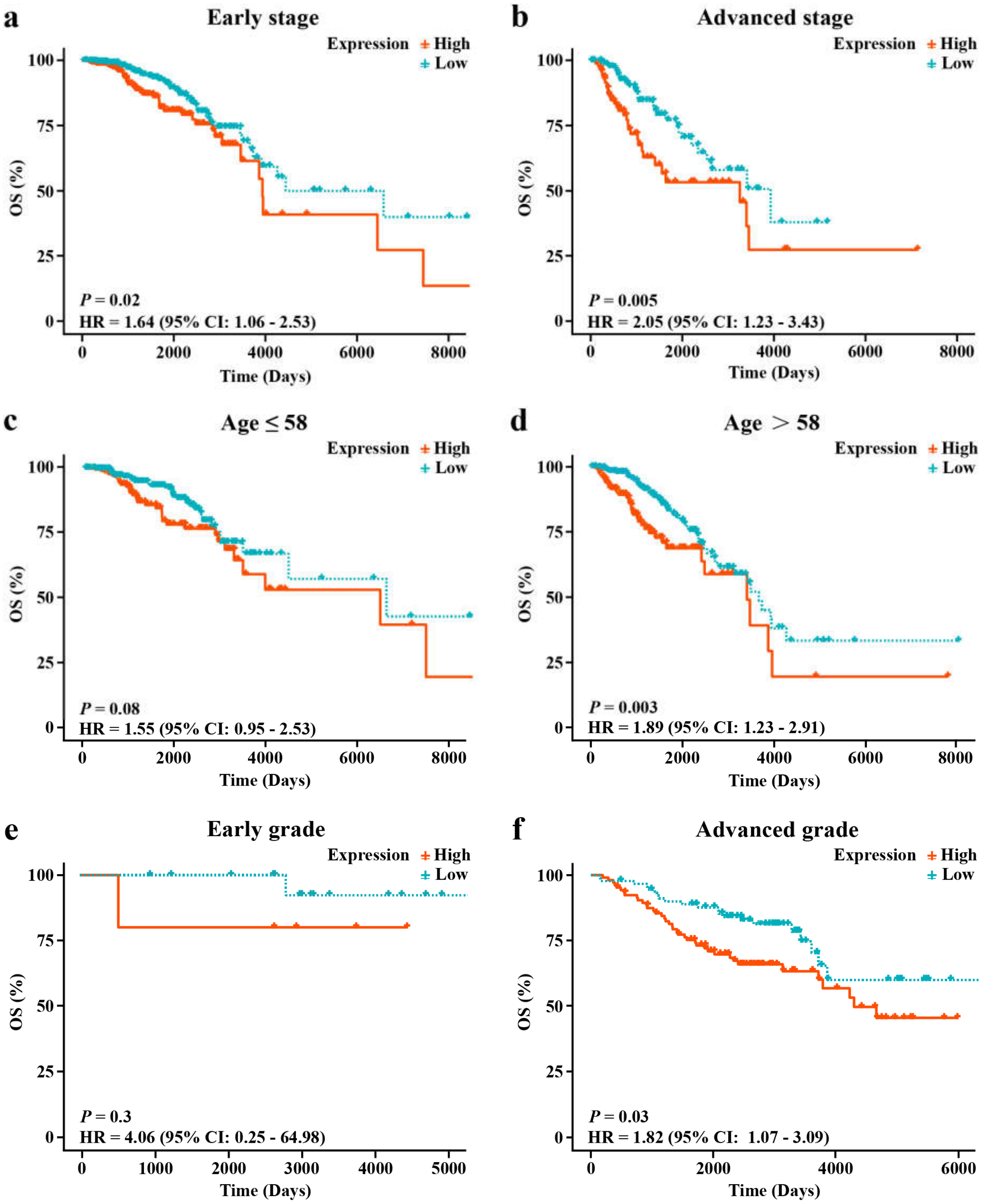

Figure S3. KM survival stratification analyses of clinical characteristics pertaining to (**a**) early stage, (**b**) advanced stage, (**c**) age ≤58, (**d**) age >58, (**e**) early grade, and (**f**) advanced grade.

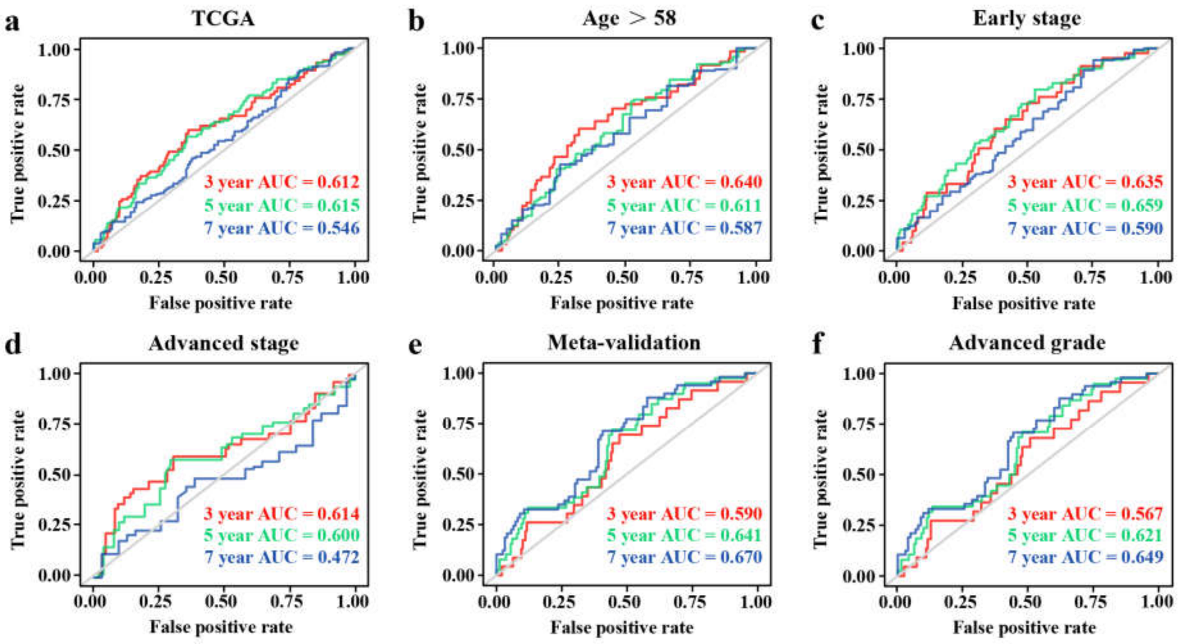

Figure S4. The time-dependent ROC curve for 3-, 5-, and 7-year survival rates. (**a**) AUC predictive value of EIF4G1 in TCGA; (**b**) AUC predictive value of EIF4G1 for age >58, (**c**) early stage, (**d**) and advanced stage in TCGA; (**e**) AUC predictive value of EIF4G1 in meta-validation; (**f**) AUC predictive value of EIF4G1 for advanced grade in GEO.
