# Supplementary material for: EIF4G1 Is a Potential Prognostic Biomarker of Breast Cancer
Source: Biomolecules. 2022 Nov 26;12(12):1756. doi: 10.3390/biom12121756 (PMC9776011; doi:10.3390/biom12121756)
Supplement: Supplementary file 1 [file biomolecules-12-01756-s001.zip › Table S2.pdf]

Table S2. Univariate and multivariate Cox analyses of prognostic factors in TCGA-training and meta-validation cohorts.

Characteristic	Univariate		Multivariate	
	HR (95% CI)	*p*-Value	HR (95% CI)	*p*-Value
**TCGA-training**				
Age	1.82 (1.32 - 2.52)	0.000285	2.21 (1.57 - 3.12)	5.48×10^{-6}
Stage	2.66 (1.90 - 3.72)	1.21×10^{-8}	2.76 (1.97 - 3.86)	3.56×10^{-9}
Risk	1.59 (1.16 - 2.19)	0.00444	1.96 (1.40 - 2.74)	7.86×10^{-5}
**Meta-validation**				
Grade	3.04 (0.95 - 9.71)	0.06050	2.46 (0.76 - 7.95)	0.13420
Risk	2.04 (1.22 - 3.41)	0.00687	1.88 (1.11 - 3.16)	0.01820

HR, hazard ratio; CI, confidence interval.
