# Supplementary material for: EIF4G1 Is a Potential Prognostic Biomarker of Breast Cancer
Source: Biomolecules. 2022 Nov 26;12(12):1756. doi: 10.3390/biom12121756 (PMC9776011; doi:10.3390/biom12121756)
Supplement: Supplementary file 1 [file biomolecules-12-01756-s001.zip › Table S3.pdf]

Table S3. Clinical data of BRCA specimens in tissue microarray.

Variable	Cases n (%)	EIF4G1 Expression		*p*-Value
		Low	High	
**Total**	80 (100%)	74	6	
**Age**				
≤ 58	60 (75.00)	56	4	0.96
> 58	20 (25.00)	18	2	
**TNM stage**				
I/II	59 (73.75)	55	4	0.18
III/IV	21 (26.25)	19	2	
**T stage**				
T1/T2	76 (95.00)	70	6	0.87
T3/T4	4 (5.00)	4	0	
**N stage**				
N0/N1	60 (75.00)	56	4	0.13
N2/N3	20 (25.00)	18	2	
**Grade**				
I/II	10 (12.50)	9	1	0.59
III	70 (87.5)	65	5	
**PR status**				
Negative	41 (51.25)	36	5	0.79
Positive	39 (48.75)	38	1	
**ER status**				
Negative	27 (33.75)	24	3	0.88
Positive	53 (66.25)	50	3	
**HER2 status**				
Negative	47 (58.75)	46	1	0.56
Positive	33 (41.25)	28	5	
**OS**				
Alive	59 (73.75)	57	2	0.58
Dead	21 (26.25)	17	4	

ER, estrogen receptor; PR, progesterone receptor; HER2, human epidermal growth factor receptor 2; OS, overall survival.
